# The influence of adjuvant on UreB protection against *Helicobacter pylori* through the diversity of CD4+ T-cell epitope repertoire

**DOI:** 10.18632/oncotarget.19248

**Published:** 2017-07-12

**Authors:** Bin Li, Hanmei Yuan, Li Chen, Heqiang Sun, Jian Hu, Shanshan Wei, Zhuo Zhao, Quanming Zou, Chao Wu

**Affiliations:** ^1^ National Engineering Research Center of Immunological Products, Department of Microbiology and Biochemical Pharmacy, College of Pharmacy, Third Military Medical University, Chongqing, PR China; ^2^ Department of Blood Transfusion, Xinqiao Hospital, Third Military Medical University, Chongqing, PR China; ^3^ Department of Intensive Care Unit, Chengdu Military General Hospital, Chengdu, PR China; ^4^ Department of Gastroenterology, Xinqiao Hospital, Third Military Medical University, Chongqing, PR China

**Keywords:** adjuvants, vaccine, protection, immunodominant, epitopes

## Abstract

Adjuvants are widely used to enhance the effects of vaccines against pathogen infections. Interestingly, different adjuvants and vaccination routes usually induce dissimilar immune responses, and can even have completely opposite effects. The mechanism remains unclear. In this study, urease B subunit (UreB), an antigen of *Helicobacter pylori* (*H. pylori*) that can induce protective immune responses, was used as a model to vaccinate mice. We investigated the effects of different adjuvants and routes on consequent T cell epitope-specific targeting and protection against *H. pylori* infection. Comparison of the protective effects of UreB, administered either subcutaneously (*sc*) or intranasally (*in*), with the adjuvants AddaVax (*sc*), Complete Freund’s adjuvant (CFA; *sc*), or CpG oligonucleotide (CpG; *sc* or *in*), indicated that only CFA (*sc*) and CpG (*in*) were protective. Protective vaccines induced T cells targeting epitopes that differed from that targeted by control vaccination. Subsequent peptide vaccination demonstrated that only two of the identified epitopes were protective: UreB_373–385_ and UreB_317–329._ Overall, we found that both adjuvant and vaccination route affected the T cell response repertoire to antigen epitopes. The data obtained in this study contribute to improved characterization of the relationship between adjuvants, routes of vaccination, and epitope-specific T cell response repertoires.

## INTRODUCTION

Vaccination is an important strategy to protect against bacteria, viruses, and other pathogens [1]; however, the development of effective human vaccines is very slow, primarily because of the limitations of available adjuvants [2]. In the past two decades, several new adjuvants have been investigated; however, their efficacy and safety in humans require further evaluation [3].

To generate robust immune responses against pathogens, it is necessary to use a potent adjuvant during vaccination. Adjuvants increase antibody titer and enhance cell-mediated immunity [3]. However, in many cases, vaccination using different adjuvants induces similar immune responses, while the protective effects are variable [4] and the mechanisms underlying this phenomenon remain unclear.

Epitopes, known as antigenic determinants, are the parts of an antigen recognized by the immune system, and are the main determinants of immune responses to entire antigens [5]. Therefore, different epitope-specific responses to the same antigen can result in varying immune responses. We hypothesized that differences in epitope-specific responses could account for observations of diverse outcomes after vaccination.

In our previous publication, urease B subunit (UreB), a protective antigen derived from *Helicobacter pylori* [6], was used to subcutaneously immunize mice in combination with Complete Freund’s adjuvant (CFA) [7] and Th1 and Th17 responses observed post-vaccination. We found that immunodominant epitope-specific Th1, but not Th17, responses mediated protection against *H. pylori* infection [7]; however, the mechanisms underlying the different protective effects observed after vaccination using different adjuvants and routes of delivery remain unclear. Therefore, in this study, UreB was administered either subcutaneously (*sc*) or intranasally (*in*) with the adjuvants, AddaVax, CFA (*sc*), or CpG oligonucleotide (CpG; *sc* or *in*), and epitope-specific Th1 responses tested post-vaccination. We found that epitope specific Th1 responses were affected by each of these different adjuvants and routes of administration, and the specific (minimal) epitopes targeted were determined. Further, the protective effects of identified epitopes were investigated. We found that the protective effects of epitopes were consistent with those in the groups vaccinated with whole antigen, combined with appropriate adjuvants. The results of different vaccination routes with the same adjuvant also reflected this phenomenon. Finally, we speculated that adjuvants may affect antigen processing by antigen-presenting cells (APCs), leading to differences in immunodominant epitopes and, hence, protective effects, following UreB vaccination with different adjuvants in the mouse model.

Together, these findings clarify that both adjuvants and vaccination routes affect the T cell response repertoire to antigen epitopes, leading to differences in protective effects post-vaccination. This knowledge could be used to design epitope-based vaccines and to predict the effects of vaccines, through detection of responses to epitopes post-vaccination, particularly in situations where this is challenging; for example, human clinical trials.

## RESULTS

### Different adjuvants and vaccination routes result in varying effects of vaccination against *H. pylori* infection

**Figure 1:**
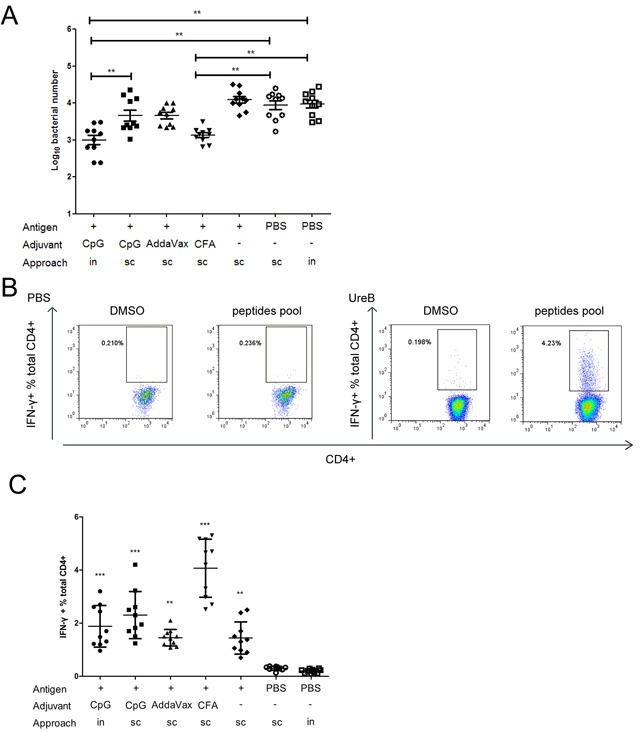
Immune responses in groups vaccinated using different adjuvants and by various routes Mice were immunized with UreB antigen in combination with different adjuvants and by various routes. Then, mice were challenged with *H. pylori*. Four weeks after the last challenge, mice were sacrificed, their stomachs harvested for evaluation of bacterial colonization, and their spleens collected for expansion of antigen-specific T cells. **(A)** Colonization of the gastric mucosa with *H. pylori* was evaluated in the different vaccination groups. **(B)** Splenic lymphocytes were isolated from PBS/UreB immunized mice post-challenge with *H. pylori* and cultured *in vitro* in the presence of UreB. On day 7, CD4+ T cells secreting IFN-γ were assessed using a UreB peptide pool. Representative flow cytometry plots demonstrating antigen-specific T cell responses from the CpG *sc* vaccination group are shown. **(C)** IFN-γ-producing CD4+ T cells from all immunized mice were assayed. All experiments were repeated three or more times. Data are expressed as means ± S. D. (n = 10). *P < 0.05, **P < 0.01, ***P < 0.001 (compared with PBS controls, unless indicated otherwise).

To determine the influences of different adjuvants and vaccination routes on vaccine effectiveness, BALB/c mice were immunized with rUreB combined with CFA (*sc*), AddaVax (*sc*), CpG (*sc*), or CpG (*in*). Then, mice were challenged with *H. pylori*. Four weeks later, mice were sacrificed and their spleens harvested for expansion of antigen-specific T cells, and bacterial colonization in their stomachs investigated. As shown in Figure 1A, *H. pylori* colonization decreased significantly in only the CpG (*in*) and CFA (*sc*) groups compared to PBS controls. Evaluation of serum IgG and mucosal sIgA antibodies indicated that they were not major contributors to the protective immune responses (data not shown). Th1 responses play a vital role in protective immunity against *H. pylori* infection [8, 9]. Therefore, antigen-specific Th1 cells from UreB immunized mice and PBS controls were expanded *in vitro* and analyzed by FACS. The specificities and purities of expanded antigen-specific T cells were confirmed to be satisfactory (data not shown). An obvious Th1 response to the UreB peptide pool was observed post-UreB immunization but not in PBS-treated controls (Figure 1B). Antigen-specific Th1 cells were induced in all vaccination groups (Figure 1C); however, the Th1 responses did not completely coincide with the extent of *H. pylori* colonization (Figure 1C and 1A), suggesting that the strength of the immune responses may not be exactly reflected in the extent of protective effects. Taken together, these results indicate that different adjuvants and vaccination routes result in varying responses to *H. pylori* infection.

### Diverse epitope-specific Th1 responses in the different vaccination groups

**Figure 2:**
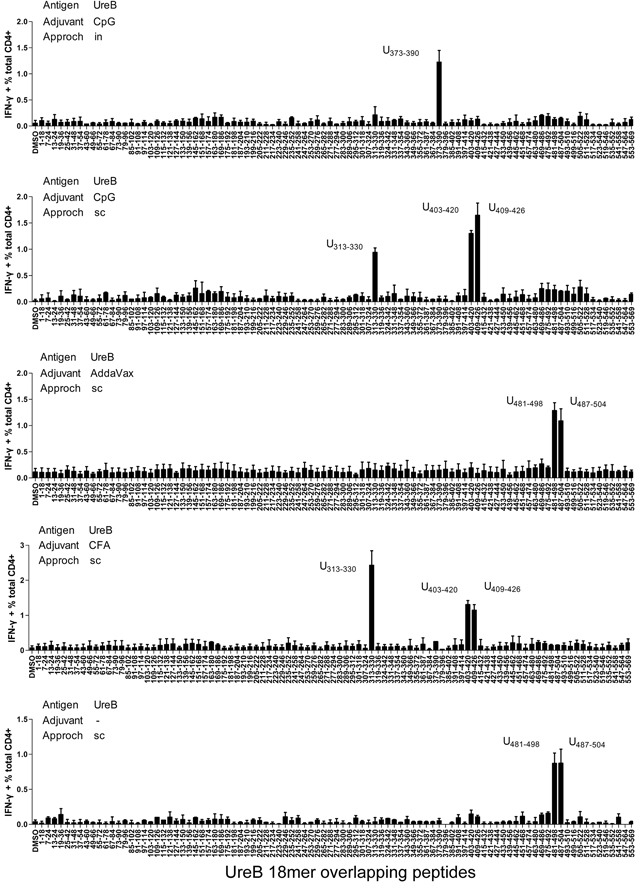
Mapping of UreB immunodominant epitopes Four weeks after *H. pylori* challenge, spleens were harvested from all mouse vaccination groups. Antigen-specific T cells were expanded *in vitro* and screened for specific IFN-γ responses to 93 overlapping 18mer peptides using ICS assays. Antigens, adjuvants, vaccination routes, and the locations of the identified 18mer peptides are indicated. The results are representative of three independent experiments. Data are expressed as means ± S. D. (n = 5).

To further investigate the T cell responses in the different vaccination groups, antigen-specific T cells were expanded *in vitro*. Then, cells were harvested and their responses against 93 individual overlapping 18mer UreB peptides screened. A total of four dominant regions of UreB were identified in all vaccination groups (Figure 2). Antigen-specific T cells from the CpG (*in*) group primarily recognized UreB_373–390_. T cells from the CpG (*sc*) and CFA (*sc*) groups had dominant responses to UreB_313–330_ and UreB_403–426_, whereas T cells from the AddaVax (*sc*) and no adjuvant groups recognized UreB_481–504_ (Figure 2). These results indicate that the immunodominant epitopes emerging in the different adjuvant and vaccination route groups varied, which could be the cause of the differing protective effects observed among the vaccination groups.

### Characteristics of immunodominant epitopes in the different vaccination groups

**Figure 3:**
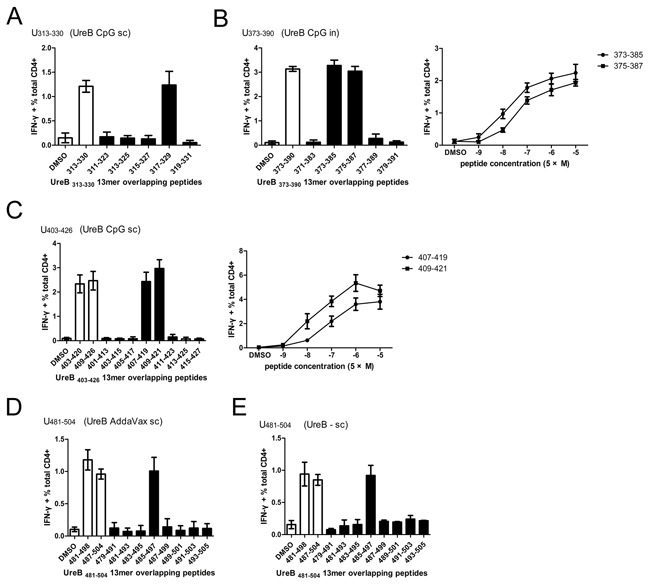
Identification of 13mer epitopes within dominant 18mer epitopes Four weeks after *H. pylori* challenge, UreB-specific T cells expanded from the spleens of immunized mice were screened for IFN-γ responses to 13mer peptides in ICS assays. **(A)** Overlapping 13mer peptides (filled bars) within the UreB_313–330_ 18mer identified as dominant in the CpG *sc* group (open bar) were tested, along with a DMSO control (open bar). **(B)** 13mer peptides within UreB_373–390_ identified in the CpG *in* group were tested, and the 13mer peptides titrated to compare their ability to activate T cell responses. **(C)** 13mer peptides within UreB_403–420_ and UreB_409–426_ identified in the CpG (*sc*) group were tested and titrated to compare their activities. **(D, E)** 13mer peptides within UreB_481–498_ and UreB_487–504_ 18mers identified in the AddaVax (*sc*) and without adjuvant control (*sc*) groups were tested. All experiments were repeated four or more times. Data are expressed as means ± S. D. (n = 5).

To further characterize the immunodominant responses induced by the 18mer peptides, splenic lymphocytes from mice immunized with UreB by different routes and combined with differential adjuvants were harvested post-*H. pylori* infection, and antigen-specific T cells expanded *in vitro*. Next, cells were harvested and stimulated with a set of overlapping 13mer peptides covering the 18mer sequences identified by screening of T cell responses. CD4+ T cells secreting IFN-γ were analyzed by ICS and FCM. For UreB_313–330_ (CpG *sc* group), immunodominant CD4+ T cells mainly recognized the 13mer peptide UreB_317–329_ (Figure 3A). As shown in Figure 3B, UreB_373–385_ and UreB_375–387_-specific Th1 responses were stronger than those of other peptides in the UreB_373–390_ 18mer peptide (CpG *in* group). Moreover, UreB_373–385_ induced more robust Th1 responses than UreB_375–387_ at different specific concentrations, suggesting that UreB_373–385_ was the main peptide responsible for induction of the immunodominant Th1 response (Figure 3B). Further, we found that UreB_407–419_ and UreB_409–421_ induced much stronger Th1 responses than other peptides within the UreB_403–420_ and UreB_409–426_ 18mers (CpG *sc* group) (Figure 3C). Peptide titration demonstrated that UreB_409–421_ was the most immunogenic peptide (Figure 3C). Antigen-specific CD4+ cells responding to the UreB_481–498_ and UreB_487–504_ 18mer peptides from the AddaVax *sc* group primarily recognized a single 13mer peptide UreB_485–497,_ indicating that UreB_485–497_ was the most immunogenic peptide within the 18mers, UreB_481–498_ and UreB_487–504_ (Figure 3D). The shorter epitope in the 18mer peptides, UreB_481–498_ and UreB_487–504_ identified in the UreB *sc* without adjuvant group was confirmed as UreB_485–497_ (Figure 3E). The 13mer overlapping peptides within the UreB_313–330_, UreB_403–420_, and UreB_409–426_ 18mers identified in the CFA *sc* group, and their MHC restriction were described in our previous study [7]. Similar data were generated from the CpG *sc* group.

**Figure 4:**
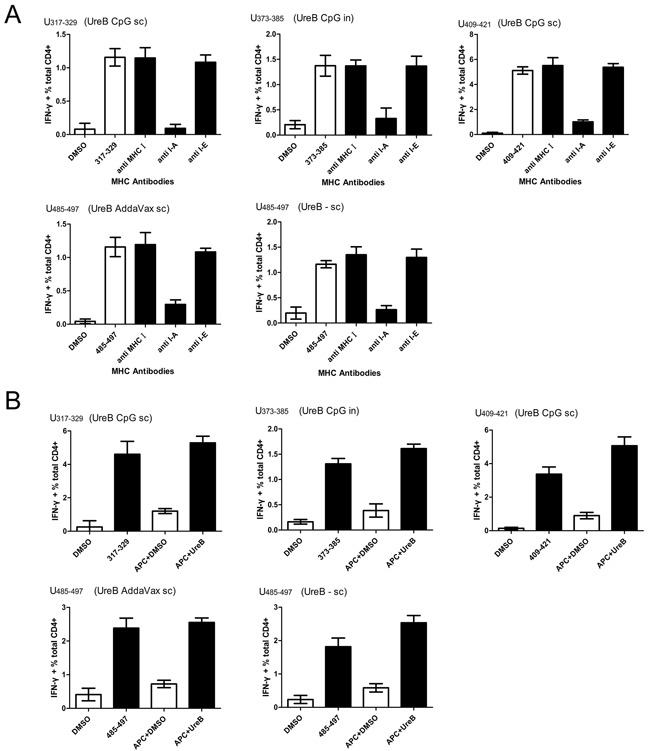
MHC restriction and natural processing and presentation of immunodominant epitopes Splenic antigen-specific T cells from immunized mice were expanded *in vitro* after *H. pylori* challenge. **(A)** To determine which MHC molecule bound to the epitopes, specific MHC antibodies were used to block MHC molecules, followed by evaluation of T cell responses. Results of DMSO control experiments and T cell responses to peptides in the absence of antibodies are shown as open bars. **(B)** Natural processing and presentation of epitopes by DCs. Results from DMSO control experiments and APCs pulsed with DMSO are shown as open bars. Antigen, adjuvants, and vaccination routes are shown. The results are representative of four or more times independent experiments. Data are expressed as means ± S. D. (n = 5).

To further confirm the MHC molecule subtypes that bound to the identified epitopes, splenic antigen-specific T cells from immunized mice post-*H. pylori* challenge were expanded *in vitro* and MHC antibody blocking assays performed. H-2^d^ (I-A) antibody effectively blocked T cell responses to epitopes UreB_317–329_ (CPG *sc*), UreB_373–385_ (CPG *in*), UreB_409–421_ (CpG *sc*), UreB_485–497_ (AddaVax *sc*), and UreB_485–497_ (UreB *sc* without adjuvant) (Figure 4A). To determine whether the immunodominant epitopes could naturally be presented by APCs, DCs were pulsed with rUreB antigen for 1 h and then cocultured with expanded epitope-specific T cells. ICS was performed to determine the abilities of these epitope-specific T cells to recognize the corresponding APCs. As shown in Figure 4B, DCs pulsed with UreB antigen effectively activated T cell responses to only their corresponding epitopes. These results indicate that the identified immunodominant epitopes could be naturally processed and presented by DCs via H-2^d^ (I-A) molecules to induce Th1 responses.

### Immunodominant epitopes in protective vaccination groups induced protective effects

**Figure 5:**
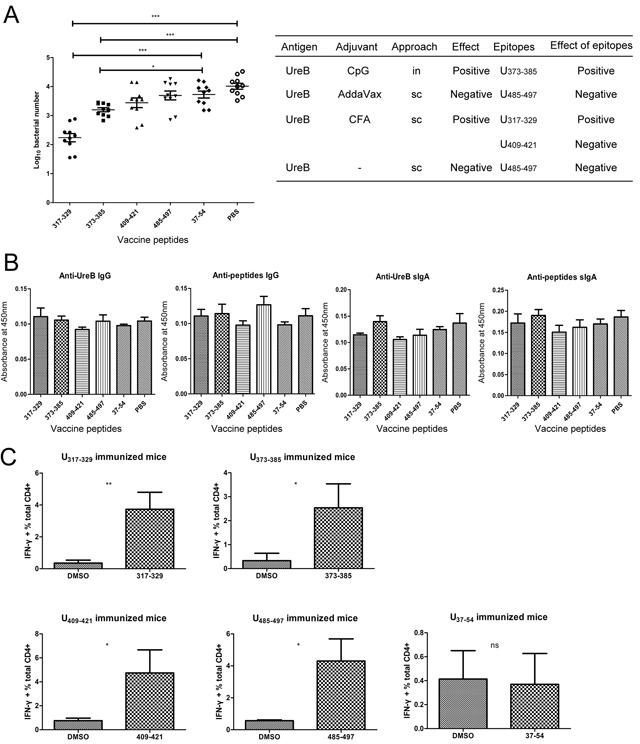
Effects of identified immunodominant epitopes in protection against *H. pylori* infection Mice were immunized by the intranasal route with the identified epitopes combined with CpG adjuvant and then challenged with *H. pylori.* **(A)** Evaluation of *H. pylori* colonization in the gastric mucosa of mice immunized with peptides. The effects of the whole protein antigen with different adjuvants and vaccination routes and the effects of corresponding epitopes are summarized to the right of the panel. **(B)** Levels of serum anti-UreB IgG, anti-peptide IgG, gastric mucosal anti-UreB sIgA, and anti-peptide sIgA antibodies were determined. **(C)** The epitope-specific T cell responses of mice immunized with peptides were analyzed. All experiments were repeated three or more times. Data are expressed as means ± S. D. (n = 10). *P < 0.05, **P < 0.01, ***P < 0.001.

To determine whether the immunodominant epitopes in the different vaccination groups could induce corresponding immune effects, mice were immunized with these epitopes combined with CpG adjuvant and challenged with *H. pylori*, followed by measurement of bacteria levels in their stomachs. As shown in Figure 5A, the levels of *H. pylori* colonization in the groups immunized with peptides UreB_317–329_ and UreB_373–385_ were much lower than those in PBS controls and those in the group immunized with the negative control peptide, UreB_37–54_. Comparison of the protective effects of vaccinations with epitopes and their corresponding antigen vaccination groups indicated that the immunodominant epitopes from the protective antigen vaccination groups also protected against infection with *H. pylori* and vice versa (Figure 5A, right). No differences in serum IgG or gastric mucosal sIgA antibodies were detected post-immunization with peptides (Figure 5B).

To assess whether peptide-specific T cell responses were activated after vaccination with peptides, splenic lymphocytes were cultured *in vitro* and their responses to the epitopes determined. As shown in Figure 5C, after vaccination with the identified immunodominant epitopes, epitope-specific T cells responses were effectively induced. Moreover, no responses were detected in mice vaccinated with the negative control peptide, UreB_37–54_.

### More robust T cell responses were induced to a protective epitope in the CFA (*sc*) vaccination group compared with the CpG (*sc*) group

**Figure 6:**
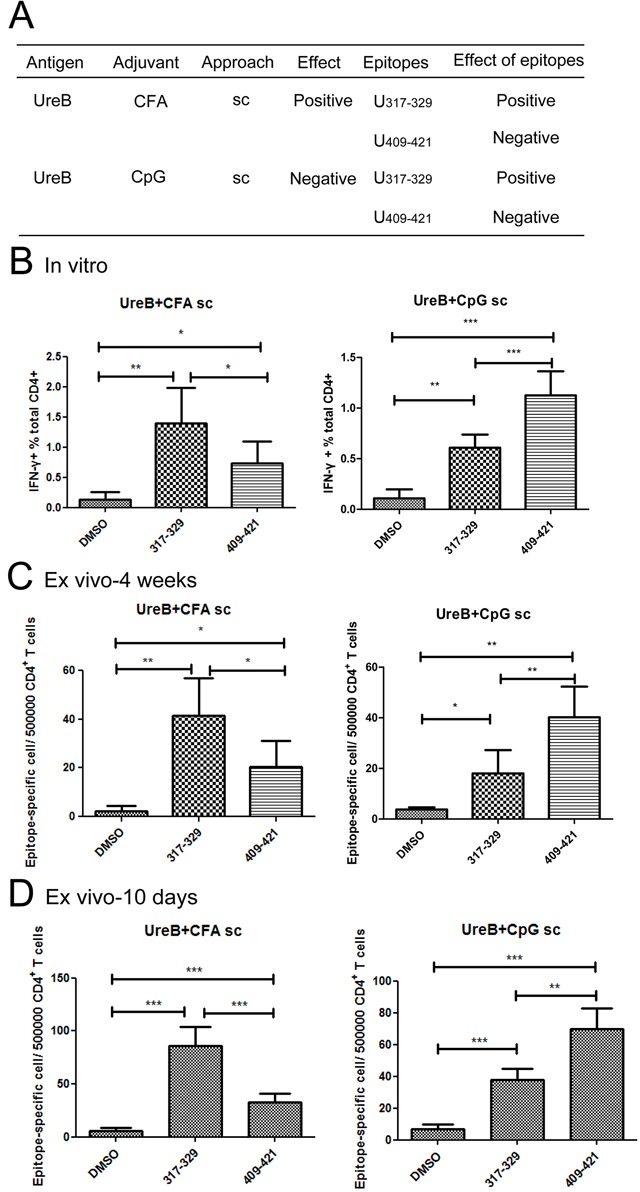
Epitope-specific T cell responses in groups vaccinated with UreB combined with CFA *sc* and CpG *sc* **(A)** The effects of vaccination with UreB combined with CFA (*sc*) and CpG (*sc*) and their corresponding epitopes are summarized. **(B)** Lymphocytes collected from mice immunized with UreB combined with CFA (*sc*) or UreB combined with CpG (*sc*) after *H. pylori* challenge were expanded *in vitro*, followed by identification of T cells responses to the dominant epitopes by FACS. **(C)** Four weeks after *H. pylori* infection, UreB_317–329_ and UreB_409–421_ peptide-pulsed DCs were used to stimulate purified CD4+ T cells from mice immunized with UreB combined with CFA (*sc*) or CpG (*sc*) for ex vivo ELISPOT assays. The numbers of epitope-specific T cells/5 × 10^5^ CD4+ T cells are presented. **(D)** UreB_317–329_ and UreB_409–421_ peptide-specific CD4+T cells from CFA (*sc*) and CpG (*sc*) vaccination groups were determined using ELISPOT assays on day10 after immunization. The numbers of epitope-specific T cells/5 × 10^5^ CD4+ T cells are presented. All experiments were repeated three or more times. Data are expressed as means ± S. D. (n = 10). *P < 0.05, **P < 0.01, ***P < 0.001.

Both the protective epitope, UreB_317–329_ and the non-protective epitope, UreB_409–421_, were identified in groups vaccinated with antigen combined with CFA (*sc*) and CpG (*sc*) (Figure 2); however, only the CFA (*sc*) group was protected against infection with *H. pylori* (Figure 1A). To investigate the reason for this, epitope-specific T cell responses were analyzed in the CFA (*sc*) and CpG (*sc*) groups. As shown in Figure 6B, after expansion *in vitro*, more T cells responded to the protective UreB_317–329_ epitope in the CFA (*sc*) vaccination group, whereas T cells in the CpG (*sc*) group mainly responded to the non-protective UreB_409–421_ epitope. To confirm this finding, CD4+ lymphocytes from spleens of groups vaccinated with antigen combined with CFA (*sc*) and CpG (*sc*) were enriched ex vivo using negative selection microbeads. Next, purified T cells were co-cultured with DCs pulsed with the dominant epitopes or dimethyl sulfoxide in ELISPOT assays. As shown in Figure 6C, four weeks after *H. pylori* challenge, the CD4+ T cell responses of the CFA (*sc*) vaccination group were mainly in response to the protective epitope, UreB_317–329_. Moreover, CD4+ T cells from the non-protective group vaccinated with UreB combined with CpG (*sc*), mainly reacted to the non-protective epitope, UreB_409–421_. Epitope-specific T cell responses in CFA(*sc*) and CpG (*sc*) vaccination groups were also determined on day 10 after the vaccination. Similar results were obtained (Figure 6D). Taken together, these results indicate that, in the non-protective vaccination group (UreB antigen combined with CpG (*sc*)), although protective epitope-specific T cells responses were induced, T cell responses primarily focused on non-protective epitopes, which may diminish the effectiveness of the vaccination.

### Adjuvants may affect the antigen processing of APCs to induce variation in immunodominant responses

**Figure 7:**
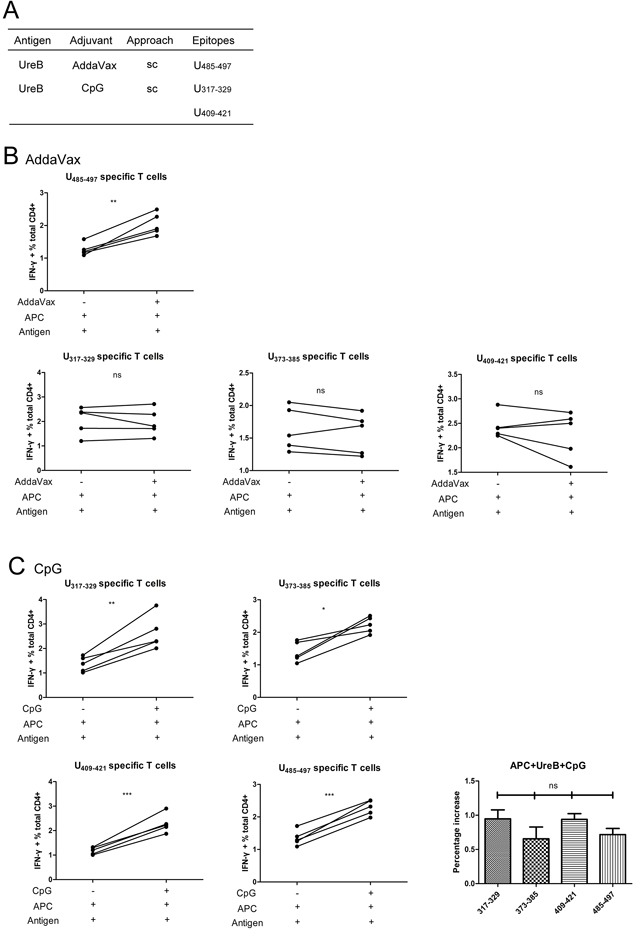
Effects of adjuvants on epitope-specific T cells responses **(A)** Summary of dominant epitopes from mice immunized with UreB combined with AddaVax (*sc*) and UreB combined with CpG (*sc*). **(B)** BM-DCs were pulsed with UreB antigen for 1 h in the presence of AddaVax. Then, these DCs were used to stimulate expanded splenic epitope-specific T cells and ICS assays performed. **(C)** DCs pulsed with UreB antigen in the presence of CpG were used to stimulate epitope-specific T cells and ICS assays performed. An increased percentage of epitope-specific T cell responses post CpG administration was demonstrated. All experiments were repeated three times. Data are expressed as means ± S. D. (n = 5). *P < 0.05, **P < 0.01, ***P < 0.001.

To investigate the mechanism underlying the induction of the various dominant epitopes by antigen vaccinations combined with different adjuvants, groups vaccinated with UreB antigen combined with AddaVax (*sc*) and CpG (*sc*) were further investigated. Although both the antigen and the vaccination route were identical in the AddaVax (*sc*) and CpG (*sc*) groups, the dominant epitopes were clearly different (Figure 7A). To determine the reason for this, BM-DCs were pulsed with UreB antigen for 1 h in the present of the adjuvants, AddaVax and CpG, separately. Then, DCs were washed and co-cultured with the four screened epitope-specific T cells. ICS was then performed to analyze the T cell responses to the pulsed DCs. As shown in Figure 7B, the responses of UreB_485–497_ specific T cells were significantly enhanced in the presence of AddaVax. No obvious differences were detected in the responses of UreB_317–329_, UreB_373–385_, and UreB_409-421_ specific T cells post AddaVax administration (Figure 7B). The effects of CpG on the responses of epitope-specific T cells were similarly determined. As shown in Figure 7C, CpG enhanced the responses of all epitope-specific T cells to DCs pulsed with antigen. Although no statistically significant differences were detected in the percentage of enhanced epitope-specific T cell responses post-CpG administration, CpG appeared to be more effective in enhancing UreB_317–329_ and UreB_409–421_ epitope-specific T cell responses (Figure 7C). Taken together, these results indicate that the adjuvants, AddaVax and CpG, may affect antigen processing and presentation in APCs.

## DISCUSSION

Adjuvants are widely used to increase immune responses to pathogen antigens and enhance the ability of vaccines to prevent infections. In addition to the traditional reason, that adjuvant can activate the innate and adaptive immune responses to poorly immunogenic vaccine antigens to induce a protective immune response, a more important motivation for incorporating adjuvants into vaccines is to achieve qualitative alteration of immune responses [10]. For example, adjuvants can direct the types of adaptive immune responses (Th1 versus Th2 [11], and T cells responses versus antibodies [12]), increase the generation of specific memory T cells, and alter the affinity of T cell receptors for peptides from pathogen antigens [13, 14]. Despite the widespread use of adjuvants, there is still no clear understanding of their cellular and molecular mechanisms of action on antigen specific T cells, particularly with regard to epitope-specific T cells [15].

In this study, the responses of epitope-specific T cells were analyzed post-vaccination with the UreB antigen of *H. pylori*, in combination with different adjuvants. CFA, which is effective in stimulating cell-mediated immunity, is widely used in experimental animal models. AddaVax emulsion is similar to MF59, which is licensed for human use in Europe [3]. Reportedly, the formulation of AddaVax promotes more balanced Th1/Th2 responses compared with those obtained using alum [16]. Although alum adjuvants are approved for use in human vaccines, they were not investigated in this study, because the main immune responses stimulated by alum are humoral immunity and Th2 responses, which are not necessary for protective immunity to *H. pylori* infection [2]. CpG, the ligand of TLR9, which induces Th1-dominated immune responses, can be administered via mucosal vaccination [17]. Therefore, in this study the three adjuvants, CFA, AddaVax, and CpG, were used to assess the protective effects of pathogen antigens. T cell response repertoires to antigen epitopes were identified post-vaccination. Although some CD4+ T cell epitopes of UreB have been published previously, the majority of these were predicted using bioinformatic algorithms [18–20], which is a rapid and economical method for epitope screening; however, it is hard to identify T cell response repertoires using bioinformatic prediction. Therefore, we synthesized a series of overlapping peptides to map immunodominant epitopes. As shown in Figure 2, antigen-specific Th1 responses to UreB were induced post-vaccination, and the immunodominant epitopes were altered by vaccination using different adjuvants and vaccination routes. Two other adjuvants, MDP and MPLA, were also used in this study and a similar phenomenon was observed (data not shown). Furthermore, the effectiveness of the epitopes identified by peptide screening in generating responses against *H. pylori* were determined, and the results confirmed that the immunodominant epitopes in the different vaccination groups contributed to the main protective response against colonization with *H. pylori* (Figure 5). Antigen-specific Th17 responses were also determined post vaccination using different adjuvants and routes in this study. But considering that the function of Th17 responses during *H. pylori* infection remains unclear, these data were not shown. Overall, the results of this study suggest that protective immunodominant epitopes induced by vaccination determine the effects of vaccines. Moreover, the adjuvant incorporated into the vaccines and the vaccination route can alter the immunodominant responses of antigen-specific T cells to these epitopes.

Vaccine adjuvants can act on APCs either directly or indirectly [21, 22]. Adjuvants induce APCs to release inflammatory cytokines, up-regulate the expression of co-stimulatory molecules, and affect the process of antigen uptake [23, 24]. Thereby, adjuvants inhibit immune tolerance of pathogens or enhance the efficacy of vaccines; however, it remains an open question whether adjuvants affect the priming of immune responses or simply enhance them [25]. APCs process antigen into peptide fragments and then display these peptides on MHC molecules on their surfaces. T cells must be activated through interaction with APCs presenting cognate peptides before they can divide and perform their function. In 1995, Vordermeier et al, demonstrated that the nature of immunogens determines the specificity of antibodies and T cells for selected peptides [26]. These authors proposed that differences in the capacity of “resting” and activated APCs (macrophage/B cells) to process and present antigen to T cells could contribute to the selection of the T cell repertoire [26]. We hypothesize that adjuvants may affect antigen processing and presentation in APCs, resulting in an alteration of the epitope repertoire and/or quantity presented. Diverse immunodominant-epitope specific T cell responses were induced after vaccinations including different adjuvants. To confirm this hypothesis, DCs derived from bone marrow were used to assess the effect of adjuvants on antigen presentation, focusing on AddaVax and CpG, since the dominant epitopes induced were clearly different in the AddaVax (*sc*) and CpG (*sc*) vaccination groups, despite the use of the same antigens and vaccination route (Figure 2). BM-DCs were separately pulsed with antigen in the presence of the two adjuvants, cocultured with epitope-specific T cells, and T cell responses detected. As shown in Figure 7, we found that AddaVax specifically enhanced the responses of UreB_485–497_ specific T cells. Although no statistically significant differences in the percentages enhanced were detected, CpG enhanced UreB_317–329_ and UreB_409–421_ epitope-specific T cells responses more effectively. CpG enhances APCs to up-regulate the expression of co-stimulatory molecules and MHC class II molecules, and increases DC maturation [27, 28], which may be the reason for the detection of enhanced responses of all epitope-specific T cells after CpG administration. Further research and additional evidence are required to verify our hypothesis. In this study, CD4+ T cell epitopes were identified post-vaccination using different adjuvants and routes; however, we believe that CD8+ T cell and B cell responses may also differ in response to vaccination with different adjuvants, and CD8+ T and B cells may have important regulatory roles in the development of the T cell repertoire [26]. Therefore, our future studies will include investigation of the roles of CD4+ T, CD8+ T, and B cell epitopes in response to antigenic peptides and the corresponding effects of adjuvants.

The route of adjuvant administration is very important in inducing effective immune responses and modulating the phenotype of T cell responses [4, 28]. For example, MF59 is a strong adjuvant for parenteral, but not for mucosal, use [29]. Moreover, after vaccination including cationic adjuvant formulation (CAF09), antigen-specific CD8+ T cell responses were elicited via intraperitoneal or intranasal administration, but not subcutaneous immunization [30]. In addition, adjuvants may induce the recruitment of DC precursors and their subsequent differentiation at the injection site and promote antigen processing and presentation [24, 27, 31]. Therefore, we believe that different vaccination routes may induce varying immunodominant epitope-specific T cell responses. Our results demonstrate that, in the CpG (*in*) group, T cells mainly responded to UreB_373–390_, whereas T cells from the CpG (*sc*) group exhibited dominant responses to UreB_313–330_ and UreB_403–426_ (Figure 2). In this study, we did not investigate the mechanisms of diverse epitope-specific T cell responses after immunization using different vaccination routes because it is difficult to harvest sufficient DCs from the intranasal mucosa or subcutaneous injection sites to allow their functional characterization. Efforts are underway in our laboratory to solve the technical problems encountered in harvesting DCs from mucosa and subcutaneous injection sites.

In this study, we used the BALB/c mouse model (which expresses MHC H-2^d^) to assess the effect of different adjuvants and routes of delivery of the UreB protein on T cell epitope-specific targeting. Therefore, the immunodominant epitopes identified in this study would likely be different to those that would be generated if other mouse strains, for example, if C57BL/6 mice (which express MHC H-2^b^), had been used. Antibody responses and inflammatory cytokines are very different among different mouse strains after vaccination with the same adjuvant [32]. Human MHC types are much more complex than those of mice; hence, every individual may exhibit unique immune response characteristics. Therefore, adjuvant-induced immune responses also differ among populations, leading to variations in vaccine efficacy [33]. Adjuvants administered to individuals and personalized vaccines may be developed in future and epitope-based vaccine experiments will facilitate their design and development.

In conclusion, UreB, an excellent vaccine candidate for protection against *H. pylori* infection, was used as a model to immunize mice in this study. Epitope-specific T cells were identified and their response repertoires analyzed post-vaccination via different routes or using vaccines containing different adjuvants. Both adjuvants and vaccination routes altered the T cell response repertoire to antigen epitopes. Moreover, the protective effects of immunodominant epitopes were consistent with those observed in groups vaccinated with the whole protein. Furthermore, we found that APCs may have an important role in this process. The data obtained in this study contribute to characterization of the relationship between adjuvants, vaccination routes, and the epitope-specific T cell response repertoire. Through the identification of immunodominant epitope-specific T cell responses post vaccination, the effects of vaccines can be predicted, particularly in situations where this is challenging; however, the data produced by this study were limited. In addition, more and more adjuvants are invented without a clear understanding of their mechanisms of action. Thus, to understand the processes by which components of vaccines modulate the immune response repertoire will require additional investigation in the future.

## MATERIALS AND METHODS

### Recombinant antigens, synthetic peptides, antibodies and adjuvants

Recombinant urease subunit B (rUreB) protein was purified and stored at −80°C. Peptides of 18mer and 13mer overlapping by 12 and 11 amino acids, respectively, were synthesized by GL Biochem (Shanghai, China) with purities of >90%. All peptides were dissolved in dimethyl sulfoxide (Sigma) and stored in aliquots at −80°C.

Anti-mouse CD3 (FITC), anti-mouse CD4 (APC), and anti-mouse IFN-γ (PE) antibodies were purchased from Biolegend. Anti-mouse MHC class I (H-2K^d^/H-2D^d^), anti-mouse MHC class II (I-A), and anti-mouse MHC class II (I-E) antibodies were purchased from eBioscience.

The adjuvants CpG OND 1826 and AddaVax were purchased from Invivogen.

### *H. pylori* culture

*H. pylori* strain B6 [34] was grown under microaerobic conditions. After 2 days, the bacteria were amplified in Brucella broth, as previously described [7]. The concentration of *H. pylori* was adjusted to 10^9^ colony-forming units (CFU)/ml prior to inoculation.

### Mice, immunization, and infection

SPF female BALB/c mice (6–8 weeks old) were purchased from the Experimental Animal Center of the Third Military Medical University. All animal experiments were approved by the Animal Ethical and Experimental Committee of the Third Military Medical University.

In the CFA group, mice were immunized with 100 μg rUreB protein emulsified in CFA subcutaneously (*sc*) in four limbs. Two weeks later, immunization was boosted with equivalent protein combined with incomplete Freund’s adjuvant. After 2 weeks, rUreB protein without adjuvant was used for the final vaccination. For the AddaVax group, mice were immunized with UreB combined with 100μl AddaVax three times using the same procedure. For the CpG subcutaneous vaccination group, mice were immunized with 100 μg UreB in 20 μg CpG three times in the same way as the AddaVax group. For the CpG (*in*) protein vaccination group and peptide immunizations, mice were immunized with 100 μg UreB or 50 μg peptides in 20 μg CpG via the intranasal route (10μl/mouse) four times with one-week intervals between vaccinations. Control mice were immunized with UreB without adjuvants, or with PBS instead of protein or peptides, using the same procedure. One week after the final boost, mice were challenged four times with 2 × 10^8^ colony-forming units of *H. pylori* by intubation.

### Determination of *H. pylori* colonization

Four weeks after the last infection, the mice were sacrificed to determine colonization with *H. pylori* in the stomach by real-time quantitative PCR [7].

### Specific T cell bulk culture and ELISAs

Four weeks after the final infection, immunized mice were sacrificed and splenic lymphocytes harvested. Then, isolated splenic lymphocytes were cultured *in vitro* and specific T cells expanded, as previously described [7]. Briefly, lymphocytes were pulsed with rUreB protein (0.5 μM) in RP-10 (consisting of RPMI-1640 (GIBCO) supplemented with 10% FCS, 2-ME (5 × 10^−5^ M) and antibiotics (penicillin 100 U/ml, streptomycin 100 μg/ml)) containing 5 U/ml rmIL-2 (PeproTech, Rocky Hill, NJ). Five days later, dead cells were removed using a Ficoll-Hypaque gradient. Next, lymphocytes were collected and cultured in RP-10 containing 20 U/ml rmIL-2.

ELISAs were performed to assess the levels of serum IgG antibodies and gastric mucosal sIgA antibodies, as previously described [34].

### Generation of dendritic cells (DCs) and co-culture with epitope-specific T cells in the present of adjuvants

Bone marrow of wild-type BALB/c mice was harvested. After treatment with erythrocyte lysis buffer, the cells were cultured in RP-10 for two hours. Then, nonadherent cells were removed and fresh RP-10 supplemented with 200 U/ml recombinant murine GM-CSF and 5 U/ml recombinant murine IL-4 added. Eight days later, nonadherent cells (DCs) were harvested and pulsed with 0.5 μM UreB antigen for 1 h in the present of the adjuvants, AddaVax (2 μl) or CpG (20 μg), in 200 μl RP-10. DCs were then washed and co-cultured with epitope-specific T cells at a ratio of 1:5 for 5 h in the presence of brefeldin A.

### Intracellular cytokine staining (ICS)

In peptide screening assays, cultured lymphocytes were incubated with peptides at 5 μM in RP-10 for 5 h in the presence of brefeldin A. In assays to determine MHC-restriction, lymphocytes were initially blocked with specific MHC antibodies for 30 min, washed and then pulsed with peptides for 5 h. Next, the cells were labeled with anti-CD3-FITC and anti-CD4-APC at 4°C for 30 min and then fixed with 4% paraformaldehyde at 4°C for 20 min. Finally, the cells were washed and labeled with anti-IFN-γ-PE in 0.2% saponin. One hundred thousand cells were acquired using a FACS Canto II flow cytometer (Becton Dickinson). FCS data were analyzed using Flowjo software.

### Ex vivo IFN-γ enzyme-linked immunosorbent spot (ELISPOT) assays

CD4+ T cells were purified from the spleens of immunized mice using CD4 negative selection MACS microbeads (Miltenyi Biotec), then ELISPOT assays were conducted using a Mouse IFN-gamma precoated ELISPOT kit (Dakewe, Beijing, China). Briefly, 5 × 10^5^ CD4+ T cells were incubated with 10^5^ peptide-pulsed DCs in microplates pre-coated with anti–IFN-γ mAb. After an overnight incubation, plates were washed and incubated with biotinylated anti-IFN-γ mAb at 37°C for 1 h. Next, the plates were washed and incubated with streptavidin-horseradish peroxidase for 1 h at 37°C. Finally, plates were washed and spots revealed using AEC Buffer. Spots were counted and the results are presented as the number of spot-forming cells/5 × 10^5^ CD4+ T cells.

### Statistical analyses

Epitope-specific T cell response data post-immunization with peptides were analyzed using unpaired *t*-tests. Data relating to the effects of adjuvants on epitope-specific T cells responses were analyzed using paired *t*-tests. Other data were analyzed using one-way ANOVA tests. All data were analyzed using the SPSS 13.0 statistical program and are expressed as means ± standard deviation (S. D.). Values of p ≤ 0.05 were considered statistically significant.
